# A Compact and Tunable Active Inductor-Based Bandpass Filter with High Dynamic Range for UHF Band Applications

**DOI:** 10.3390/s25103089

**Published:** 2025-05-13

**Authors:** Sehmi Saad, Fayrouz Haddad, Aymen Ben Hammadi

**Affiliations:** 1 Technopôle de Château Gombert, Aix Marseille Université, IM2NP UMR 7334, 13397 Marseille, France; 2 Dynamics RF/mmW Consulting and Design, 39 avenue du Vercors, 38600 Grenoble, France

**Keywords:** bandpass filter, differential active inductor, UHF, wide tuning range, CMOS

## Abstract

This paper presents a fully integrated bandpass filter (BPF) with high tunability based on a novel differential active inductor (DAI), designed for sensor interface circuits operating in the ultra-high frequency (UHF) band. The design of the proposed DAI is based on a symmetrical configuration, utilizing a differential amplifier for the feedforward transconductance and a common-source (CS) transistor for the feedback transconductance. By integrating a cascode scheme with a feedback resistor, the quality factor of the active inductor is significantly improved, leading to enhanced mid-band gain for the bandpass filter. To facilitate independent tuning of the BPF‘s center frequency and mid-band gain, an active resistor adjustment and bias voltage control are employed, providing precise control over the filter’s operational parameters. Post-layout simulations and process corner results are conducted with 0.13 µm CMOS technology at 1.2 V supply voltage. The proposed second order BPF achieves a broad tuning range of 280 MHz to 2.426 GHz, with a passband gain between 8.9 dB and 16.54 dB. The design demonstrates a maximum noise figure of 16.54 dB at 280 MHz, an input-referred 1 dB compression point of −3.78 dBm, and a third-order input intercept point (IIP3) of −0.897 dBm. Additionally, the BPF occupies an active area of only 68.2×30 µm^2^, including impedance-matching part, and consumes a DC power of 14–20 mW. The compact size and low power consumption of the design make it highly suitable for integration into modern wireless sensor interfaces where performance and area efficiency are critical.

## 1. Introduction

The rapid expansion of wireless communication systems has significantly transformed modern technological fields, driven by the increasing demand for high-speed, reliable, and scalable data transmission. The frequency range between 300 MHz and 3 GHz represents a crucial spectrum for a diverse set of radio frequency (RF) applications, owing to its favorable propagation characteristics and versatility.

This frequency range supports critical applications across communication, navigation, industrial, and consumer domains [1], and plays an essential role in enabling a broad spectrum of sensor-based systems and wireless sensing applications. In smart wireless communication systems, frequencies within this band are extensively employed in applications such as cellular networks [2], industrial telemetry [3,4], and experimental wireless platforms [5,6], many of which integrate embedded sensing capabilities for real-time data acquisition, environmental monitoring, and system diagnostics.

Cellular bands (e.g., 700 MHz, 850 MHz, 1.8 GHz, and 2.1 GHz) constitute the foundation of modern internet of things (IoT) and machine-type communication (MTC) infrastructures, supporting various sensor-driven applications including smart metering, asset tracking, remote health diagnostics, and infrastructure condition monitoring. Industrial telemetry systems operating within the UHF band (e.g., 400–470 MHz) are widely deployed for remote sensing of pressure, temperature, and vibration in utility grids, manufacturing environments, and energy facilities. Furthermore, the globally unlicensed industrial, scientific, and medical (ISM) band at 2.4 GHz enables widely adopted short-range communication protocols such as Wi-Fi, Bluetooth, BAN, Wireless HART, Z-wave, and ZigBee, which are the foundation of wireless sensor networks (WSNs) used in building automation, smart agriculture, healthcare wearables, and industrial IoT systems. Experimental sensor deployments have also leveraged amateur radio bands (e.g., 433 MHz, 902–928 MHz), particularly in educational and research contexts for applications like remote weather stations, aerial sensing with balloons and drones, and emergency telemetry. This frequency range also supports sensor-integrated systems in navigation and radar applications. For instance, global navigation satellite systems (GNSS) operating in the L-band (1.2–1.6 GHz) provide high-precision positioning for sensors in autonomous vehicles, drones, and geodetic instruments. Similarly, weather and maritime radar systems in the S-band (2.7–3.0 GHz) deliver real-time environmental data essential for climate monitoring, air traffic control, and disaster early warning systems. Additionally, low-power wide-area networks (LPWANs) operating in sub-GHz bands (e.g., 433 MHz, 868 MHz, and 915 MHz) offer long-range, low-energy solutions for distributed sensing networks in smart cities, logistics, environmental sensing, and precision farming [7]. The military and aerospace sectors further exploit this spectrum for tactical sensor networks, including target tracking, surveillance, and secure telemetry systems.

**Figure 1:**
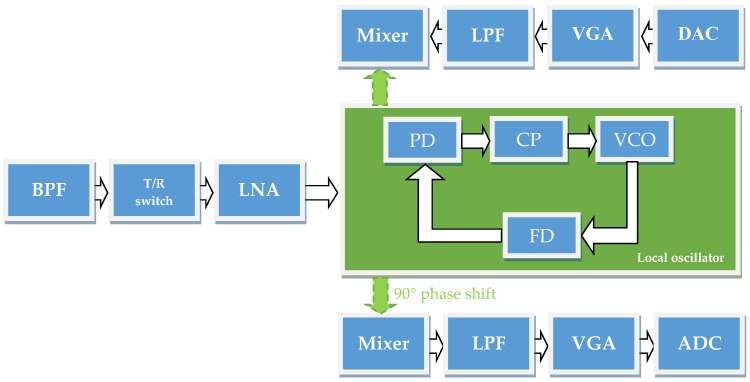
Transceiver front-end circuit.

With the ongoing development of wireless communication technology, the design of reconfigurable RF integrated circuits (ICs) has become a crucial focus area for next-generation wireless sensing platforms. One of the most essential components of every wireless transceiver is the bandpass filter (BPF). Figure 1 presents the transceiver front-end circuit positioning a BPF as the first component in the processing chain. The BPF is directly integrated with the antenna without the inclusion of intermediary components, ensuring seamless and unimpeded interaction with the received signal. Upon reception, the antenna transmits the sensed signal to the BPF, which either selectively passes the desired frequency range or attenuates unwanted signals outside the target bandwidth. This filtering process ensures the integrity of the signal passed to the subsequent processing stages.

Consequently, the performance characteristics of the BPF, such as its bandwidth, insertion loss, and out-of-band rejection, significantly influence the overall performance of both the receiver and the transmitted signal [8].

Several integrated RF-BPF filter topologies have been reported in the literature, including MOSFET-C filters [9], active-RC filters [10], switched-capacitor filters [11,12], Gm-C filters [13], and Q-enhanced LC filters [14]. Each filter’s configuration suffers from one or more limitations that restrict their use in gigahertz-frequency integrated applications. The continuous-time filters based on MOSFET-C filters and active-RC integrators have limited high-frequency operation due to the finite gain-bandwidth product (GBWP) of the operational amplifiers (op-amps) used in the feedback circuit. Meanwhile, switched-capacitor filters suffer from clock feedthrough problems.

Among these, Gm-C and Q-enhanced LC filters have shown greater promise for implementation at high frequencies. Q-enhanced LC topology utilizes lossy LC-tank resonators and improves their effective Q-factor through negative resistance compensation circuits. However, their practical implementation at RF frequencies is hindered by challenges such as the bulky nature of inductors, limited scalability, moderate quality factors of on-chip monolithic inductors (typically below 12) [15], and various parasitic losses, including ohmic losses, substrate-induced eddy currents, and resistive losses. Furthermore, these filters suffer from limited dynamic range and tunability, as well as a dependency on the Q-factor of the tank inductors, which directly impacts overall filter performance. Efforts to improve the Q-factor of monolithic inductors (Q < 40) using fabrication process modifications have shown some promise. However, such methods often increase manufacturing complexity and costs, and reduce the reliability of large-scale production [16,17,18].

To overcome the challenges associated with monolithic inductors in standard silicon processes, active inductors (AIs) have become increasingly preferred by many RF circuit designers, owing to their numerous advantageous properties. Compared to monolithic inductors, active inductors offer several advantages, including a high and tunable quality factor, a higher self-resonance frequency, and a reduced chip area.

Active inductors can be categorized into three primary types: amplifier-based AIs [19,20], current conveyor-based AIs [21,22], and gyrator-C AIs [23]. The first two types require many passive and active components, which often result in larger area requirements. Additionally, these AIs are associated with drawbacks such as limited tunability, significant power dissipation, and a constrained frequency range. On the other hand, gyrator-C-based AIs provide several advantages, including a higher Q-factor, greater self-resonance frequency, improved tunability, and reduced size [24].

Various studies have introduced techniques to enhance the performance of gyrator-C-based active inductors. These methods target improvements in key parameters, such as the operating frequency range, tunability, Q-factor, power efficiency, and linearity. Leveraging a cascode or regulated cascode configuration [25,26] in the feedback path enhances the Q-factor and extends the frequency range of AIs by suppressing zero frequency. Exploiting a feedback resistor technique [27,28] leads to an increase in Q-factor and inductance simultaneously. Flipped and cascode-flipped [29,30,31], additive capacitor [32], DC level shifter [33], double feedback [34], and distortion reduction [35] are among the various techniques reported in the literature to enhance the performance of AIs. Class AB active inductors were introduced in [36,37]. However, these inductors consume significant power and are inherently noisy.

For optimal performance in radio frequency applications, differential active inductor structures are superior to single-ended designs due to their wider dynamic range and higher quality factor [38]. Single-ended AIs lack symmetry, resulting in inconsistent port characteristics that deviate from ideal inductor behavior. Despite progress, many discussed active inductor designs in literature fail to simultaneously achieve desired key specifications, such as compactness, low power consumption, high Q-factor, tunability, and low noise. Thus, creating a robust active inductor that integrates these features into a single, efficient design remains a critical challenge.

This paper presents a novel DAI with a focus on its 2nd-order tunable BPF implementation for UHF band applications using 0.13 µm CMOS technology. By incorporating several design techniques, the DAI achieves enhanced performance metrics, including an improved Q-factor, broader frequency range, and greater tunability. The proposed differential structure distinguishes itself from existing designs in the literature. To validate its effectiveness, a reconfigurable BPF is designed and simulated. This filter achieves a wide center frequency range from 280 MHz to 2.426 GHz, enabling compatibility with various multi-standard, sensor-based communication modules such as LTE, GSM, Bluetooth, Wi-Fi, ZigBee, LoRa, and others. The transistors used in our design were sized and biased in the saturation region following the g_m_/I_D_ methodology.

The remainder of this paper is structured as follows. Section 2 describes the basic concept of a gyrator-C-based active inductor and describes the design of the novel DAI. Section 3 deals with tunable second-order BPF design using the proposed DAI in Section 2. Section 4 focuses on the simulation results of the BPF, along with a performance comparison to other reported works, where process—voltage—temperature (PVT) analyses are also presented. Section 5 concludes and discusses the proposed work.

## 2. Differential Active Inductor Design

### 2.1. Basic Concept of Differential Active Inductors

To create a floating active inductor, the key principle is to design a circuit that satisfies the short-circuit admittance matrix equation for a two-port network,

(1) $$\left[\begin{array}{c} I_{1}\\ I_{2} \end{array}\right]=sL_{eq}\left[\begin{array}{cc} 1 & -1\\ -1 & 1 \end{array}\right]\left[\begin{array}{c} V_{1}^{+}-V_{1}^{-}\\ V_{2}^{+}-V_{2}^{-} \end{array}\right]$$

where L_eq_ is the equivalent inductance value of the floating active inductor.

**Figure 2:**
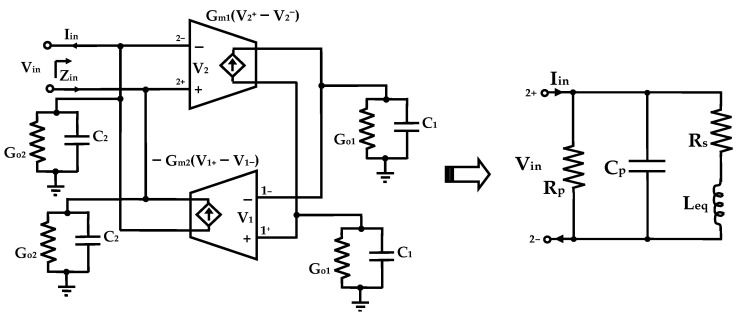
Schematic of a lossy DAI based on gyrator-C networks and its equivalent RLC circuit.

A gyrator can be created by incorporating two operational transconductances amplifier (OTA) connected back to back in single loop. One OTA is positioned in the feedback-path, which provides a negative transconductance and another in the forward path, which provides a positive transconductance. When a capacitor is connected to the output of a gyrator, the resulting configuration is known as a gyrator-C network. This network leverages the intrinsic properties of the gyrator to transform the capacitive impedance into an inductive impedance, effectively emulating the behavior of a physical inductor. Figure 2 depicts the structure of a lossy differential gyrator-C network.

KCL equations at nodes 2+ and 2− show that the input admittance Y_in_ can be given as follows:

(2) $$Y_{in}=\frac{I_{in}}{V_{2}^{+}-V_{2}^{-}}=s\frac{C_{2}}{2}+\frac{G_{o2}}{2}+\frac{1}{s\frac{C_{1}}{2G_{m1}G_{m2}}+\frac{G_{o1}}{2G_{m1}G_{m2}}}$$

where g_o1_, and g_o2_, are the total conductances at node 1 and node 2, respectively.

Since the input/output impedances of the transconductors in the gyrator-C network are inherently limited, the synthesized equivalent inductor exhibits a lossy behavior. Equation (2) is characterized by the presence of parasitic components, including resistance and capacitance, which degrade the ideal inductive response. The behavior of the lossy inductor can be modeled by the equivalent RLC network, as illustrated in Figure 2, and its equivalent circuit parameters are derived from the small-signal analysis of the gyrator-C network. These parameters are calculated as follows:

(3) $$C_{p}=\frac{C_{2}}{2},\;R_{p}=\frac{2}{G_{o2}},\;L_{eq}=\frac{C_{1}/2}{g_{m1}g_{m2}}\mathrm{and}\;R_{s}=\frac{G_{o1}/2}{g_{m1}g_{m2}}$$

where R_p_ is the parallel resistance, C_p_ is parallel capacitance, R_s_ is the series resistance, and the L_eq_ equivalent inductance.

The corresponding resonance frequency and Q-factor are given by matching the real and imaginary parts equations

(4) $$\omega_{0}=\frac{1}{L_{eq}C_{p}}=\sqrt{2\frac{g_{m1}g_{m2}}{C_{1}C_{2}}}$$

(5) $$Q\approx \left(\frac{\omega L_{eq}}{R_{s}}\right)\left(\frac{R_{p}}{R_{p}+R_{s}\left[1+{\left(\frac{\omega L_{eq}}{R_{s}}\right)}^{2}\right]}\right)\left[1-\frac{R_{s}^{²}C_{p}}{L_{eq}}-\omega^{²}L_{eq}C_{p}\right]$$

Due to the presence of parasitic components, originating from the intrinsic characteristics of MOS transistors, the input admittance is not purely inductive behavior over all the entire frequency range. To achieve low ohmic losses, it is essential to maximize the parasitic parallel resistance R_p_, thereby reducing energy dissipation through parallel leakage paths, and to minimize the parasitic series resistance R_s_, which reduces resistive losses and enhances the overall performance.

To overcome the loss limitations, a feedback resistance is frequently employed to mitigate the parasitic series resistance, which adversely affects the Q-factor of the active inductor [39]. This approach introduces a parallel effect on both the series resistance and the inductance. Consequently, the feedback resistance simultaneously decreases the series loss resistance and enhances the emulated inductance L_s_, theoretically driving Q toward infinity. Moreover, the feedback mechanism serves to fine-tune the phase of the input admittance and the self-resonant frequency (SRF), thereby optimizing the active inductor’s operating frequency at which Q achieves its maximum value.

A regulated cascode configuration is also used by many designers to take advantage of the benefits of series-connected transistors, as described in [40]. This approach effectively solved the main limitation of the active inductor, which arises from the conductance g_o1_ at node 1. By adopting the cascode topology, the quality factor can be significantly improved and help reduce both the power consumption and supply voltage requirements of the active inductor. Furthermore, the transconductance of other transistors can be used to change the emulated inductive value.

Indeed, active inductors involve a tradeoff between SRF, equivalent inductance, power consumption, and Q-factor. To achieve low power consumption and a high SRF, a smaller input transistor is required. Conversely, a larger input transistor is essential for attaining a higher quality factor and greater inductance value, highlighting the design compromises necessary for optimizing performance [41].

### 2.2. Implementation of the Proposed Differential Active Inductor

**Figure 3:**
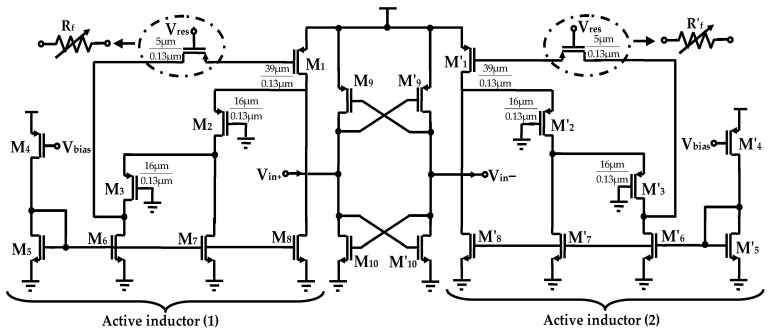
Proposed differential active inductor circuit.

The configuration of the proposed differential active inductor, computed using the design custom method, is shown in Figure 3. It comprises two grounded active inductors coupled through two cross-coupled transistor pairs that form negative resistances. Each inductor consists of three transistors M_1_, M_2_, M_3_ for AI-1, and M_15_, M_16_, M_17_ for AI-2, providing the basic inductance functionality.

Two controllable current mirror sources are employed for polarization, modeled by transistors M_18_ to M_22_ for active inductor (1) and M_4_ to M_8_ active inductor (2). To improve the Q-factor and the inductance value of each active inductor, cascode stages, implemented with transistors M_3_ and M_17_, are utilized as gain-boosting stages. These cascode configurations effectively reduce the output conductance g_ds_ of transistors M_2_ and M_16_, respectively, leading to enhanced performance. Furthermore, two controllable feedback resistors, R_f_, are incorporated to optimize the Q-factor while introducing a negative conductance to compensate for the parasitics and losses inherent in the AIs. These resistors are implemented using PMOS transistors M_9_ and M_23_, biased in their ohmic regions. The negative resistance is generated through a double cross-coupled PMOS/NMOS differential pair M_10_, M_24_ and M_11_, M_25_, which delivers a negative resistance of −2/g_m_, where g_m_ represents the transconductance of each cross-coupled transistor.

The operation of the tunable active inductor is as follows. When a differential input voltage is applied to the gates of the common-source transistors M_1_ and M_15_, the transconductances g_m1_ and g_m15_ convert the voltage into a drain current, charging the gate capacitances C_gs2_ and C_gs17_ of transistors M_2_ and M_17_, respectively. The voltages developed across C_gs3_ and C_gs16_ are subsequently converted into input currents by the transconductances of transistors M_3_ and M_16_, respectively, emulating the voltage-current characteristics of a shunt inductance.

**Figure 4:**
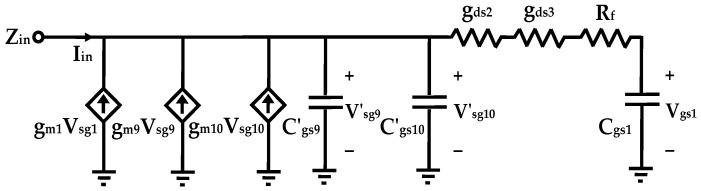
Equivalent small signal model of the proposed differential active inductor.

Due to the symmetry of the circuit topology, the simplified small-signal equivalent half-circuit is presented in Figure 4.

By assuming C_dsi_ ≪ C_gsi_, the input admittance Y_in_ can be derived as:

(6) $$Y_{in}=\frac{1}{Z_{in}}=-\frac{g_{m9}}{2}-\frac{g_{m10}}{2}+\frac{R_{f}+r_{ds9}+r_{ds10}}{2}+\frac{sC_{gs1}+sC_{gs9}^{\prime }+sC_{gs10}^{\prime }}{2}+\frac{g_{m}}{2\left(1+\left(R_{f}+r_{ds9}+r_{ds10}\right)sC_{gs1}\right)}$$

where C_gsi_ is the gate-source capacitance, and g_mi_ and g_dsi_ are the transconductance and output conductance of the i-th MOS transistor, respectively. The equivalent RLC model parameters of the DAI are derived as follows:

(7) $$L_{eq}=\frac{2C_{gs1}\left(R_{f}+1/g_{ds2}+1/g_{ds3}\right)}{g_{m1}}$$

(8) $$C_{p}=\frac{C_{gs1}+C_{gs9}^{\prime }+C_{gs10}^{\prime }}{2}$$

(9) $$G_{p}=\frac{-\left(g_{m9}+g_{m10}\right)}{2}+\frac{R_{f}+1/g_{ds2}+1/g_{ds3}}{2}$$

(10) $$R_{s}=\frac{2}{g_{m1}}$$

The inclusion of a feedback resistance R_f_ introduces a positive contribution to both the resistance and inductance in the RLC equivalent model of the proposed active inductance. By carefully selecting the value of R_f_, the equivalent inductance L_eq_ can be significantly enhanced, while the parallel resistance R_p_ can be maximized. Furthermore, maintaining a fixed transconductance for transistor M_1_ effectively reduces the equivalent series resistance R_s_ of the active inductor. Additionally, L_eq_ can be independently tuned by adjusting R_f_ or modifying the conductance of other transistors, providing enhanced flexibility in circuit optimization.

The self-resonant frequency of the DAI, defined as the frequency at which the imaginary part of the input admittance becomes zero, can be calculated as:

(11) $$\omega_{0-DAI}\approx \frac{1}{\sqrt{L_{eq}C_{p}}}=\frac{1}{\sqrt{\frac{R_{f}+r_{ds2}+r_{ds3}}{g_{m1}}\left(C_{gs1}^{2}+C_{gs9}^{\prime }+C_{gs10}^{\prime }\right)}}$$

The Q-factor of the DAI is defined as the ratio of the real part to the imaginary part of the input admittance Y_in_ and is expressed as:

(12) $$Q_{DAI}=\frac{ℑ(1/Y_{in})}{ℜ(1/Y_{in})}\approx \frac{R_{p}}{\omega L_{eq}}=\left|\frac{g_{m1}}{\omega C_{gs1}\left(R_{f}+r_{ds2}+r_{ds3}\right)\left(R_{f}+r_{ds2}+r_{ds3}-g_{m9}^{\prime }-g_{m10}^{\prime }\right)}\right|$$

where $g_{eq}=1/\left(R_{f}+r_{ds2}+r_{ds3}\right)$.

Based on Equation (12), by selecting R_f_ + r_ds2_ + r_ds3_ to be approximately equal to − g_m9_′ − g_m10_′, the parallel resistance R_p_ approaches infinity. Consequently, the Q-factor at frequencies below the self-resonant frequency can become significantly large.

According to the above equations, the characteristics of the proposed DAI can be independently adjusted with minimal mutual interference. Variations in bias currents provide an additional mechanism for controlling both the inductance L_eq_ and the Q-factor Q_DAI_. By varying the transconductance, an additional capacitance is introduced into the node described in Equation (6), offering further tuning capabilities for the proposed circuit.

## 3. Proposed Tunable Bandpass Filter

### 3.1. Bandpass Filter Design Methodology

**Figure 5:**
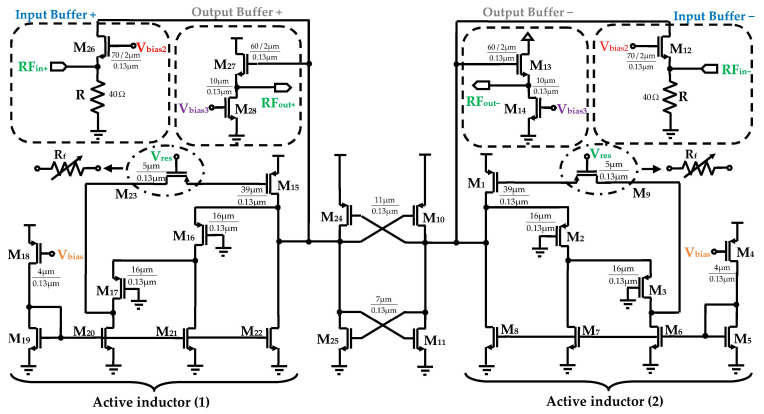
Proposed DAI based bandpass filter.

In this section, the implementation of an RF bandpass filter using the proposed DAI is presented, with the intent of improving filter performance and minimizing chip area. The circuit diagram of the differential BPF including the compensation and tuning circuitry is illustrated in Figure 5, where a second order filter architecture was designed. It incorporates the required degrees of freedom to facilitate tuning the desired center frequency, bandwidth, noise figure, and power consumption.

The circuit comprises four RF stages, with the first stage being a differential input buffer. This buffer converts the differential input RF voltages, V_RFin+_ and V_RFin−_, into a current, which is then applied to the RLC network represented by the tunable DAI.

This block is implemented using two common source-follower NMOS transistors, M_12_ and M_26_, connected to two resistors R. The second stage, which forms the filter core, comprises the AIs connected via two cross-coupled negative resistances. The negative resistance reduces resistive losses, thereby increasing and controlling the Q-factor of the circuit. The third stage is a differential output buffer configured in a source-follower topology, consisting of transistors M_13_ and M_27_, along with two controlled gate transistors, M_14_ and M_28_. This stage is designed to drive the resistive load while preventing the load from significantly degrading the BPF’s performance. Device dimensions, i.e., width W, length L, as well as the number of fingers Nf, of all the devices (M_1_ through M_26_) for the simulation of the proposed DAI and BPF are reported in Figure 5. A multi-finger MOSFET layout technique [41,42] was employed to optimize these parameters, aiming to improve performance while minimizing parasitic effects, reducing power consumption and ensuring better matching and layout efficiency [43].

For optimal matching of input and output impedance, the transistors in the input buffer M_12_ and M_26_ were sized with a W of 70 μm, L of 0.13 μm, and Nf = 2, after optimization. To achieve input-impedance matching of 50 Ω, a resistor R with a value of 40 Ω is incorporated, eliminating the need for additional capacitors or inductors. In the output stage, a second impedance-matching circuit is crucial to ensure maximum transfer power and to mitigate the impact of the load on the filter’s frequency response and quality factor. To match the output impedance to 50 Ω, a 600 fF metal-insulator-metal (MIM) capacitor is integrated into the output buffer. Furthermore, the output stage transistors M_13_ and M_27_ are sized with a W of 60 μm and Nf = 2. The gate-to-source voltage V_gs_ of the cascode stages (M_2_, M_3,_ and M_16_, M_17_) is set to ground (GND) to simplify the design.

The resonant frequency can be adjusted by varying the control voltages V_res_ and V_bias2_. The resonant frequency of the BPF is expressed as:

(13) $$\omega_{BPF}=\frac{1}{\sqrt{L\left(C_{p}+C_{par}\right)}}$$

where C_p_ ≈ C_gs1_ + C_gs1_′, and C_par_ represents the parasitic capacitance contributed by the input C_gd26_ and output buffers C_gs27_. Considering the assumption C_gd_ ≪ C_gs_, the parasitic capacitance is approximated as C_par_ = C_gs27_ + C_gs27_′.

### 3.2. Theoretical Analysis

**Figure 6:**
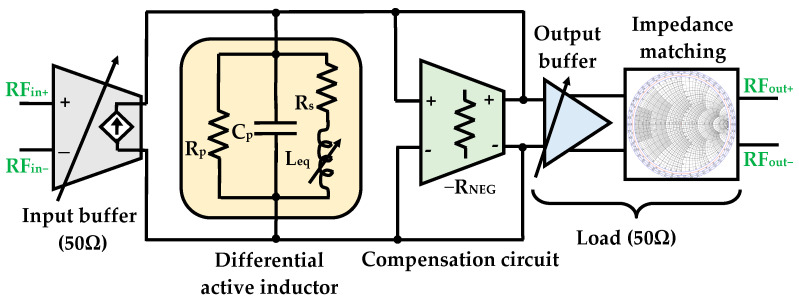
Configuration block of a DAI-based BPF.

Figure 6 illustrates the configuration of the bandpass filter, including the RLC equivalent network of the DAI. To minimize the equivalent parasitic capacitance and resistance in the active inductor, a negative impedance transformation −R_NEG_ is employed. Referring to Figure 6, the gain-bandwidth (GBW) of the bandpass filter, derived from the overall model, can be approximated as:

(14) $$A_{2}=\frac{g_{m}}{G_{p}-G_{NEG}}$$

Therefore, the Q-factor of the BPF is obtained as:

(15) $$Q_{BPF}=\frac{\omega_{0-DAI}}{BW}=\frac{1}{\left(G_{p}-G_{NEG}\right)L_{eq}\omega_{0-DAI}}=Q_{DAI}\frac{1/R_{p}}{1/R_{p}-1/R_{NEG}}$$

In order to achieve optimal compensation condition, the parallel resistance R_p_ must equal the magnitude of the negative resistance ∣R_NEG_∣, whereas the stability condition of the BPF is satisfied when G_p_ > ∣R_NEG_∣.

## 4. Simulation Results and Discussion

The proposed DAI and resulting BPF were designed and implemented using 130 nm CMOS technology from STMicroelectronics. The circuit operation and performance was evaluated using the Virtuoso ADE^©^ environment from Cadence IC.6.1.9^©^, with parasitic effects taken into account. A comprehensive set of simulations was performed to evaluate the performance parameters and their robustness under various operating conditions. These simulations encompassed DC analysis, S-parameter evaluation, noise characterization, periodic steady-state (PSS) analysis, process corner evaluation, and Monte Carlo tests, ensuring a thorough assessment of the design’s behavior across diverse environments. It is worth noting that all simulation results and graphical data were obtained using Cadence Virtuoso and subsequently processed and visualized using Origin Lab^©^. The simulated performance results of the proposed active BPF topology are summarized in Table 1.

### 4.1. Differential Active Inductor Performances

In the first phase of the analysis, the performance of the proposed differential active inductor was evaluated by analyzing its key characteristic parameters, including input impedance Z_in_, effective equivalent inductance L_eq_, Q-factor Q_DAI_, and self-resonance frequency.

**Figure 7:**
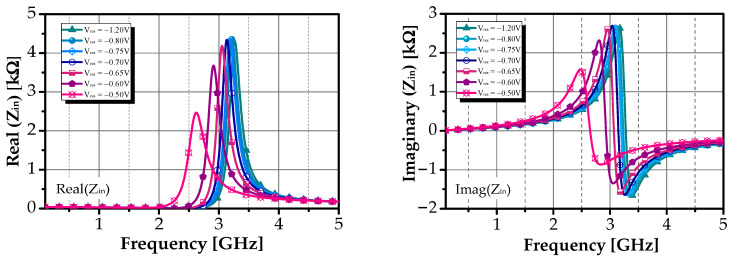
The real and imaginary parts of the proposed active inductor’s tuning for different V_res_.

The input impedance Z_in_ was composed of a resistive component *ℜ*{Z_in_} and an inductive reactance *ℑ*{Z_in_}, which were determined using a two-port S-parameter analysis. The frequency dependence of Z_in_, *ℜ*{Z_in_}, and *ℑ*{Z_in_} for the differential active inductor is depicted in Figure 7. Additionally, the inductive behavior of the proposed DAI was verified by analyzing the phase response under varying R_f_. According to [44], the emulated inductance value L_eq_ and Q-factor Q_DAI_ can be derived from the input impedance Z_in_ as follows:

(16) $$Z_{in}=Z_{11}-Z_{21}-Z_{12}+Z_{22}$$

(17) $$Q_{DAI}=\frac{\left|ℑ\left(Z_{in}\right)\right|}{ℜ\left(Z_{in}\right)}\;\;L_{eq}=\frac{ℑ\left(Z_{in}\right)}{\omega }$$

The variation in R_f_ is achieved by adjusting V_res_ from −0.5 V to −1.2 V. Under these conditions, L_eq_ was found to range between approximately 33 nH and 98 nH. Additionally, the circuit exhibited self-resonance frequencies of up to 3.3 GHz.

**Figure 8:**
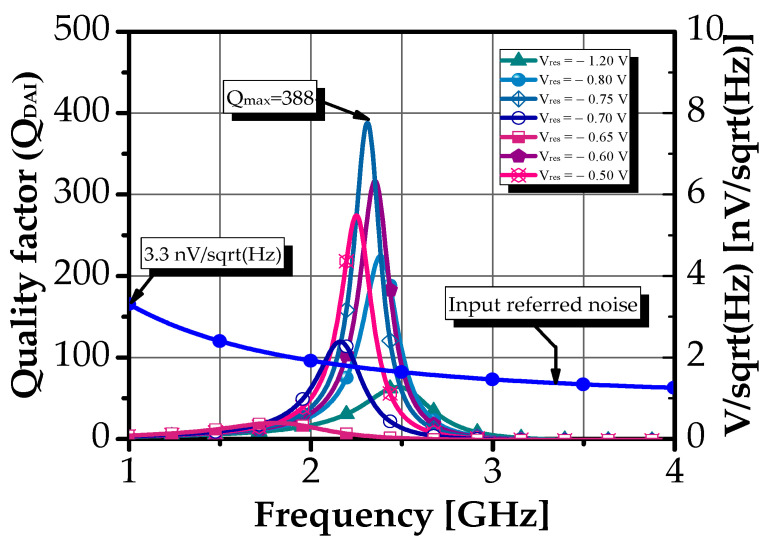
Q-factor value versus frequency over V_res_ and input-referred noise voltage of the proposed DAI.

As illustrated in Figure 8, Q_DAI_ was optimized to exceed 20 across the entire operating frequency band. Notably, a peak quality factor of approximately 388 was achieved at 2.31 GHz when V_res_ was set to −0.7 V. This high degree of tunability in the Q-factor is a significant advantage of the proposed design.

**Figure 9:**
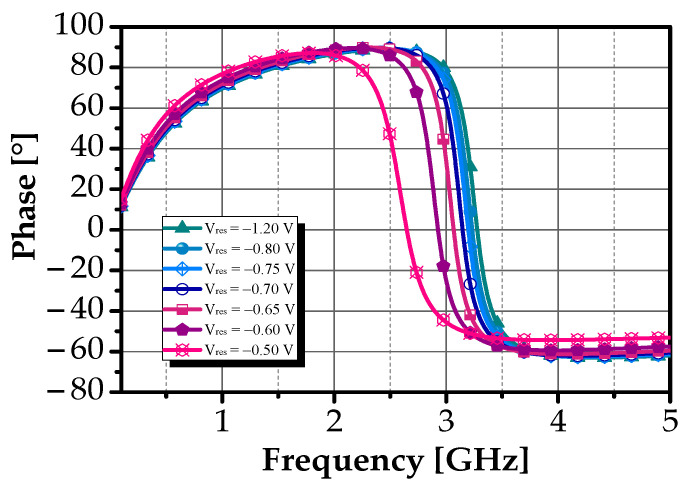
Phase response of the proposed DAI over V_res_.

To further validate the inductive behavior of the circuit, a detailed analysis of its phase characteristics was conducted. As shown in Figure 9, when V_res_ was set to −0.7 V, the phase approached 89.85° across the entire operating frequency range, confirming that the circuit maintained its inductive behavior within this band. For frequencies beyond 3.3 GHz, the reactance component of Z_in_ becomes negative, indicating a transition to capacitive behavior.

The linearity of the differential AI has been investigated in prior studies [45,46]. Intermodulation tests were conducted using a two-tone input signal at 1.997 GHz and 2 GHz, yielding an input third-order intercept point (IIP3) of −9.71 dBm. The 1 dB compression point (P_1dB_) for the proposed DAI was determined to be 2.25 dBm, corresponding to a voltage swing of 0.82 V at the inductor’s differential input with a 1.2 V supply voltage.

### 4.2. Bandpass Filter Performances

To achieve a wide frequency tuning range, two control voltages were utilized. The first control voltage was adjusted by regulating V_res_, which controls the feedback transistors M_9_ and M_23_. The second control voltage was implemented through controllable current sources transistors M_12_ and M_26_ via V_bias2_. These control mechanisms were calibrated to minimize the noise figure while achieving high gain and low power consumption. Note that V_res_ was employed for coarse frequency tuning, whereas V_bias2_ enabled fine frequency adjustments. The supply voltage remained fixed at 1.2 V, with the tuning range of V_res_ and V_bias2_ varying from −1.2 V to 0.2 V, and from 0.5 V to 0.8 V, respectively. Meanwhile, the bias voltages V_bias_ and V_bias3_ were fixed at 0.5 V, providing an optimal compromise between power consumption and compensation.

**Figure 10:**
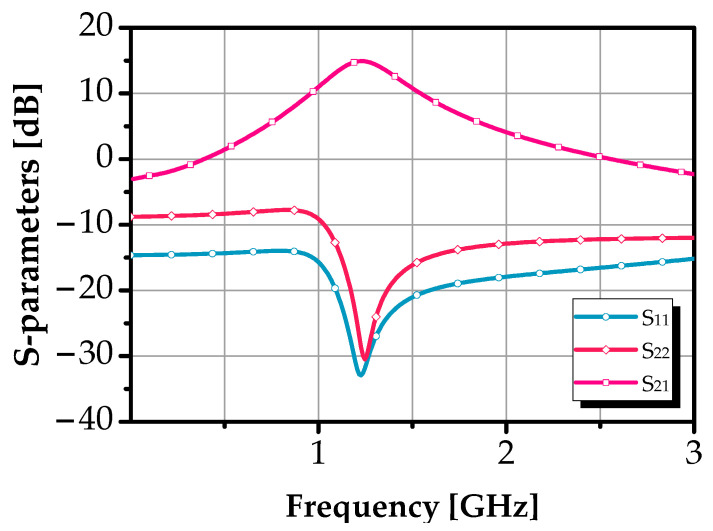
Simulation response of S-parameters of the proposed BPF.

In Figure 10, the simulated S-parameters of the filter are presented under its fourth tuning state (see Table 1), providing valuable insight into the circuit’s performance. This piece of detail is usually missing in the state-of-the-art of active BPF. However, their behaviors were imported for evaluating the stability characteristics and the input and output impedance matching of the design. The proposed BPF is centered at 1.228 GHz and exhibits an input reflection coefficient (S_11_) and an output reflection coefficient (S_22_) of −33 dB and −31 dB, respectively, indicating excellent impedance matching and optimal power transfer across the filter’s passband.

At this center frequency, the filter reached a 3-dB BW of approximately 400 MHz, which aligns with the expected behavior of a 2nd-order active BPF in the literature. In contrast, various previously reported 2nd-order BPFs utilizing active inductors achieve narrow 3-dB BW by incorporating a high value of negative series resistance. Although this technique effectively reduces the bandwidth, it often introduces practical stability concerns and significantly increases the risk of oscillation. Therefore, to ensure reliable operation, an active inductor must always maintain unconditional stability throughout its intended operating range.

**Figure 11:**
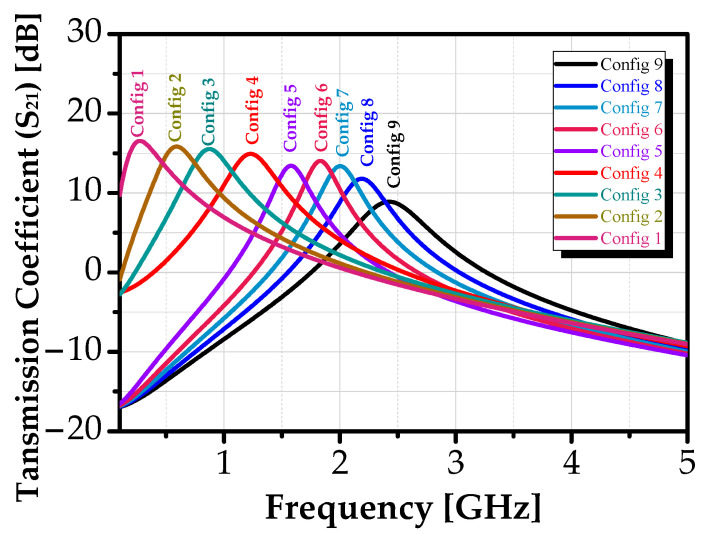
Transmission coefficient S_21_ of the proposed BPF for different combinations of [V_res_, V_bias2_].

Figure 11 illustrates the variation in the transmission coefficient *S*_21_ response. The results demonstrate that the center frequency *f*_c_ can be effectively tuned from 0.28 GHz, to 2.426 GHz, by adjusting the values of V_res_ and V_bias2_ according to the configuration settings listed in Table 1, spanning Config 1 to Config 9. A maximum forward transmission coefficient (S_21_) of 16.54 dB was achieved at the center frequency of 0.28 MHz, i.e., Config 1, indicating optimal signal transmission.

The resulting −3 dB bandwidth (BW) of the proposed design ranged from 287 MHz to 406 MHz. Additionally, the medium gain of the filter enabled it to achieve an effective Q-factor of 6.4 at a center frequency of 1.83 GHz.

**Figure 12:**
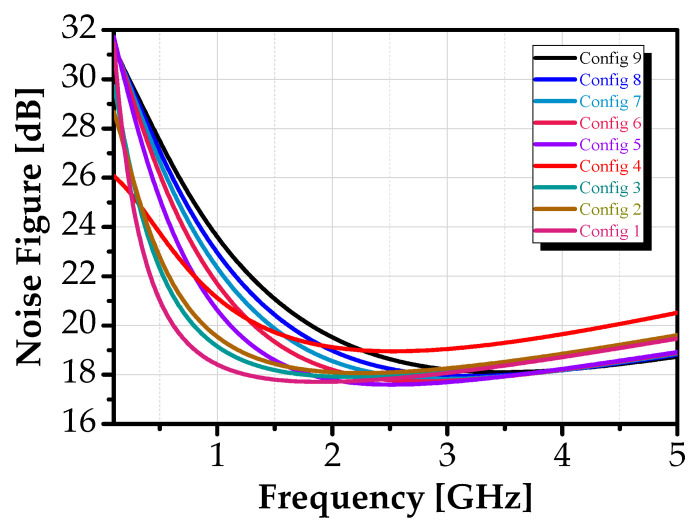
Noise figure of the proposed BPF.

A comprehensive noise analysis of the proposed topology was performed, including its dynamic range, evaluated in terms of the noise figure (NF). Figure 12 depicts the NF as a function of frequency, revealing that the NF varied from 18.41 dB at 1.57 GHz to 24.6 dB at 280 MHz. In comparison to other active bandpass filters reported in the literature [47,48], these NF values remain well-suited for practical applications.

The results obtained for various combinations of control voltages V_res_ and V_bias2_ are summarized in Table 1.

**Figure 13:**
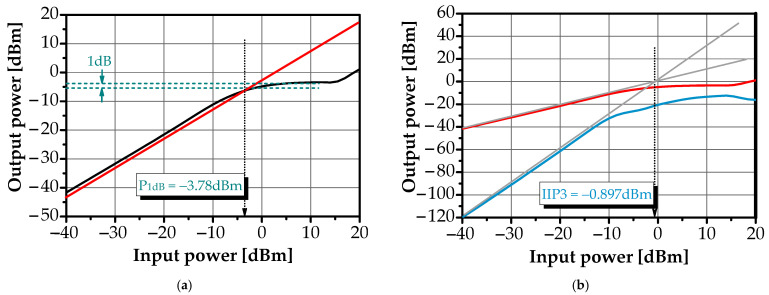
(**a**) 1-dB compression point, (**b**) input referred third-order of the proposed BPF.

The linearity of the BPF was evaluated using the 1 dB compression point (P_1dB_) and the third-order intercept point (IIP3). To assess its performance, two-tone signals with a spacing of ±2.5 MHz were applied. As shown in Figure 13a,b, the P_1dB_ distortion point was achieved at an input power of −3.78 dBm, while the IIP3 was measured at approximately −0.897 dBm. These results demonstrate that the proposed active filter exhibits high linearity, making it well-suited for multiband applications in integrated circuit design.

**Figure 14:**
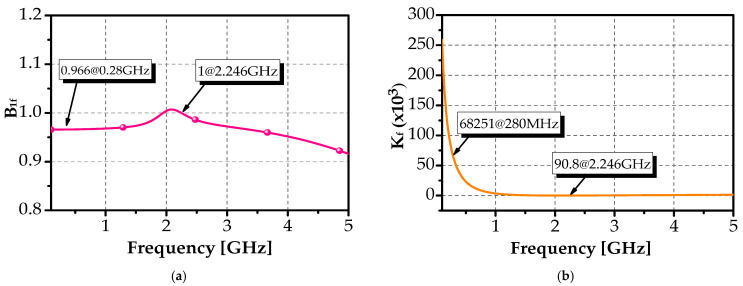
Stability properties of the proposed BPF (**a**) alternative stability factor B_1f_, (**b**) Rollet stability factor K_f_.

In this part, the analysis results of the proposed design regarding alternative stability factor B_1f_ and Rollet stability factor K_f_ responses are depicted in Figure 14.

As defined by Equations (11) and (12), a two-port circuit is considered unconditionally stable if B_1f_ > 0 and K_f_ > 1.

(18) $$B_{1f}=1-{\left|\Delta \right|}^{2}+{\left|S_{11}\right|}^{2}-{\left|S_{22}\right|}^{2}>0$$

(19) $$K_{f}=\frac{1-{\left|\Delta \right|}^{2}+{\left|S_{11}\right|}^{2}-{\left|S_{22}\right|}^{2}}{2\left|S_{11}\right|\left|S_{21}\right|}>1$$

From the plot shown in Figure 14, the stability analysis of the proposed filter confirms a sufficient stability margin across the operational frequencies and beyond, ensuring an unconditionally stable design. Specifically, B_1f_ was approximately 0.966 (greater than 0), while K_f_ remained consistently greater than 1 over the entire frequency band.

### 4.3. Monte Carlo Analysis

In the nanoscale area, several factors contribute to the degradation of MOS device performance [49,50], including oxide thickness variation (OTV), metal-gate work-function fluctuations (WKF), random dopant fluctuations (RDF), and line-width roughness (LWR).

To investigate these phenomena and analyze their impact on circuit performance, process-sensitive device parameters such as doping concentration, channel width W, channel length L, and gate oxide thickness t_ox_ were incorporated into a Monte Carlo simulation analysis. The variability in the bandpass filter’s forward transmission coefficient S_21_, noise figure NF, Rollet stability coefficient K_f_, and alternative stability factor B_1f_ was estimated using 1000 Monte Carlo simulation runs. This approach ensured a reduction in the standard deviation (σ) of the estimated values to below 5%, enhancing the accuracy of the variability analysis.

Table 2 summarizes the sensitivity analysis under mismatch and process variation obtained from statistical histograms. The Gaussian distributions at ±3δ level obtained from the Monte Carlo analysis indicate that approximately 99.7% of the simulated values for the forward gain S_21_ fell within the range of 13.67 to 15.94 dB, in distribution of the mean value (µ), i.e., 14.9 dB. Similarly, NF maintained a stable mean of 19.94 dB with a majority of samples concentrated between 19.81 and 20.04 dB, reflecting excellent consistency. Furthermore, K_f_ and B_1f_ parameters exhibited average values of 9.82 and 0.998, respectively, with corresponding variations of 4.93% and 0.09%. Both metrics consistently remained well above the critical stability thresholds throughout all Monte Carlo runs, indicating that the circuit maintained unconditional stability even under worst-case mismatch and process variation scenarios. The Monte Carlo analysis demonstrates that the proposed circuit exhibited tightly bounded variation under mismatch and process fluctuations, confirming its robustness, high reliability, and suitability for stable, high-yield integration in practical CMOS-based RF systems.

### 4.4. Process Corner Analysis

**Figure 15:**
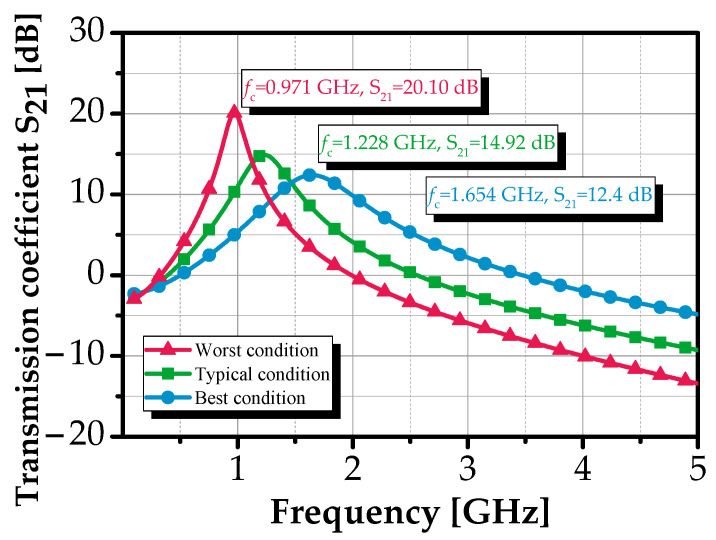
Performance variation of transmission coefficient S_21_ of the proposed BPF with various corner conditions.

Figure 15 illustrates the forward transmission coefficient *S_21_*, which maintained a value of 14.9 dB under three process variation scenarios: typical NMOS-typical PMOS (TT), fast NMOS-fast PMOS (FF), and slow NMOS-slow PMOS (SS). The best-case scenario occurred under the FF corner at 1.1 V and a temperature of −40 °C, while the worst-case scenario was observed under the SS corner at 0.9 V and a temperature of +125 °C.

The forward transmission coefficient S_21_ of the filter maintained an acceptable value (>12 dB) even under varying conditions, ensuring it remained sufficient for the target BPF’s gain. However, the center frequency *f*_c_ demonstrated significant sensitivity to voltage and temperature variations, exhibiting a shift of approximately 34% across all process corners. This degradation is primarily attributed to fluctuations in the reference current. These variations can be effectively mitigated by adjusting the bias current generator or incorporating a temperature-compensated current reference [51]. This underscores the robustness of the proposed design for integration into wireless communication devices and systems.

### 4.5. Layout and Performance Comparison

**Figure 16:**
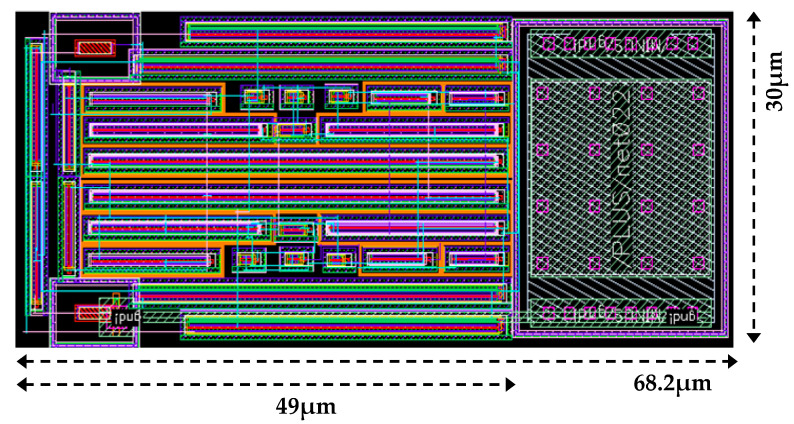
Layout of the proposed BPF.

The layout of the proposed BPF is depicted in Figure 16. It occupies a total area of 2046 µm^2^ (30 µm × 68.2 µm), including the output-matching impedance capacitor. The circuit’s DC power consumption ranges between 14.1 mW and 20.3 mW, operating at a nominal supply voltage of 1.2 V.

The design metrics of the proposed BPF are summarized in Table 3 and compared against the state-of-the-art active BPFs reported in the literature. The dynamic range (DR) of the bandpass filter, as defined in [48], is expressed as:

(20) $$DR=\frac{P_{1dB}}{P_{n}}=\frac{P_{1dB}}{kTF}$$

where P_1dB_ represents the input 1-dB compression point in dBm, and P_n_ is the filter’s noise power relative to a 1-Hz bandwidth, given by P_n_ = kT. Here, kT (thermal noise floor) is −174 dBm/Hz and F is the noise factor.

To evaluate and compare the performance of bandpass filters, a figure-of-merit (FoM) was utilized, where a higher FoM indicates superior filter performance. Two widely adopted definitions of FoM, as detailed in [52,53,54,55], are given by:

(21) $$FoM_{1}=\frac{1}{P_{DC}}\frac{P_{1dB}}{P_{n}}$$

and

(22) $$FoM_{2}=\frac{P_{1dBW}{𝑓}_{BPF}Q_{BPF}N}{P_{DC}NF}$$

where P_1dB_ and P_1dBw_ denote the 1-dB compression points in dBm and watts, respectively, P_DC_ is the DC power consumption in mW, P_n_ is the noise power corresponding to a 1-Hz bandwidth in dBm, Q_BPF_ is the quality factor, f_BPF_ is the resonant frequency of the filter, N is the number of poles, and NF is the noise figure (expressed as a unit less ratio rather than in decibels).

Table 3 highlights that the proposed bandpass filter (BPF) achieved a first figure of merit (FoM_1_) of 140.3 dB-Hz/mW, positioning it among the top-performing designs. Although slightly lower than those reported in [39,56], the difference is primarily attributed to the lower power consumption achieved in those works. Nonetheless, the proposed architecture demonstrated outstanding performance, including the widest tuning range and smallest chip area among the compared works, at an expense of a moderate noise figure. Furthermore, the design maintained reasonable power consumption, reinforcing its overall efficiency and competitiveness. The second figure of merit (FoM_2_), which evaluated frequency selectivity, power efficiency, linearity, and noise performance, reached 73 dB, making it comparable to existing implementations in the literature.

**Table 3:** Performance comparison inductor-based BPF.

	Reference	[39]	[55]	[56]	[57]	[58]	[59]	Our Work
Parameter								
Filter order (N)		2	2	2	2	2	1	**2**
Inductor Topology		Gyrator-C active inductor						
SRF (GHz)		1.16–3.27	5.15–5.35	1.6–2.9	1–2	3.02–3.76	0.1–1.3	**0.28–2.426**
−3dB BW (MHz)		65	15	80	1000	-	300	**294–406**
V_DD_ (V)		1.0	1.2	1.8	1.2	20	-	**1.2**
Inductor Q		300–794	964	-	-	12	-	**388**
Gain S_21_ (dB)		26.62–33.45	15	5.75	0.5	−12.2–13.7	0	**8.89–16.54**
P_DC_ (mW)		4.04–6.44	7.71	6	30	506	120	**14.1–20.3**
P_1dB_ (dBm)		2.72	−2.8	−7	−21	−11.65	5	**−** **3.78**
NF (dB)		14.48–16.56	21.7	6	19	N/A	11	**18.41–24.60**
DR (dB-Hz)		161.7 ^a^	149.5 ^a^	161 ^a^	134 ^a^	-	82	**151.8**
FoM_1_ (dB-Hz/mW)		153.9 ^a^	140.6 ^a^	153.1 ^a^	119.2 ^a^	-	61.2 ^a^	**140.3**
FoM_2_ (dB)		92.7 ^a^	92.2	91 ^a^	41.8 ^a^	-	64 ^a^	**73**
Area (mm^2^)		0.017	-	0.023	0.18	0.062	-	**0.002 ^b^**
Sim./Meas.		Sim.	Sim.	Sim.	Meas.	Meas.	Meas.	**Sim.**
Process Technology		130 nm CMOS	40 nm CMOS	180 nm CMOS	130 nm CMOS	0.5 µm pHEMT GaN	N/A	**130 nm CMOS**

^a^ Calculated based on data available in article. ^b^ Including impedance matching circuit.

## 5. Conclusions

In this paper, a reconfigurable and a fully differential active inductor has been introduced for UHF band applications, enabling the implementation of a robust tunable second-order bandpass filter using 130 nm CMOS technology with a 1.2 V supply voltage. Implementing feedback resistor and cascode transistor elements enhanced the quality factor of the active inductor, ensuring superior performance and reliability. The post-layout simulation results demonstrate that the proposed BPF design achieved a wide frequency tuning range from 280 MHz to 2.246 GHz, enabled by two control voltages. The design further exhibited high linearity, good selectivity, low power consumption, and a compact layout, making it a highly efficient and practical solution for advanced RF and sensor-oriented applications.

Future advancements in active inductor design present substantial opportunities for enhancing filter performance in sensor interface circuits. While the use of a cascode structure and series feedback resistor successfully minimizes losses and improves the quality factor, these features may introduce stability issues and limit the tunability of inductance, especially in high-frequency scenarios. Adjusting the current bias magnitude could address these challenges and optimize performance across a wide range of conditions. However, increasing the bias current leads to higher power consumption, while lowering it may result in greater losses due to elevated series resistance R_S_. In future sensor-based systems, a focus on managing distortion under high input power conditions will be crucial. The nonlinearity of the inductance, arising from the limited transconductance in the feedback path, will need to be addressed. This nonlinearity could also restrict performance in low-voltage, high-swing environments, where stacked transistors must operate in strong inversion to meet high-frequency demands. Employing level shifters in such cases could provide an effective solution, ensuring that stacked devices in the feedback path function reliably. Overcoming these challenges and balancing the associated trade-offs will be critical in satisfying the demands of next-generation sensor technologies.

## Figures and Tables

**Table 1:** Simulated design metrics of the proposed BPF.

Config	V_res_ (mV)	V_bias2_ (mV)	*f*_c_ (GHz)	S_21_ (dB)	BW (MHz)	Q_BPF_	NF (dB)	P_DC_ (mW)
1	200	600	0.28	16.54	321	0.9	24.60	15.2
2	130	600	0.59	15.84	357	1.7	21.86	15.2
3	100	580	0.87	15.52	370	2.35	19.63	14.9
4	100	500	1.22	14.92	406	3	20.19	14.1
5	−200	800	1.57	13.42	297	5.3	18.41	20.3
6	−250	800	1.83	14.02	287	6.4	18.48	20.3
7	−300	800	2	13.38	318	6.29	18.55	20.3
8	−400	800	2.19	11.78	397	5.52	18.63	20.3
9	−1200	800	2.426	8.89	593	4.1	18.73	20.3

**Table 2:** Monto Carlo analysis: sensitivity of the proposed BPF to mismatch and process variations.

Type	S_21_ (dB)	NF (dB)	K_f_	B_1f_
Mean (µ)	14.9	19.94	9.82	0.998
Standard deviation (σ)	0.345	0.379	0.484	0.000875
Variability (σ/Mean)	2.32%	1.9%	4.93%	0.09%
Minimum (−3σ)	13.67	19.81	8.6	0.997
Maximum (+3σ)	15.94	20.04	11.6	1.002

Total number of iterations/hits = 1000, evaluated at V_res_ = 0.1V and V_bias2_ = 0.5V.

## Data Availability

Data are contained within the article.

## Abbreviations

The following abbreviations are used in this manuscript:

BPF	Bandpass Filter
DAI	Differential Active Inductor
UHF	Ultra-High Frequency
CS	Common Source
CMOS	Complementary metal-oxide-semiconductor
IIP3	Third-Order Input Intercept Point (IIP3)
DC	Direct Current
RF	Radio Frequency
IoT	Internet of Things
MTC	Machine-Type Communication
ISM	Industrial, Scientific, and Medical
Wi-Fi	Wireless Fidelity
BAN	Body Area Network
HART	Highway Addressable Remote Transducer
ZigBee	Zonal Intercommunication Global Standard
LPWAN	Low-Power Wide Area Networks
WSN	Wireless Sensor Network
GNSS	Global Navigation Satellite System
IC	Integrated Circuit
LNA	Low-Noise Amplifier
LPF	Low Pass Filter
VGA	Variable Gain Amplifier
DAC	Digital Analog Converter
ADC	Analog Digital Converter
PD	Phase Detector
CP	Charge Pump
VCO	Voltage Controlled Oscillator
FD	Frequency Divider
MOSFET	Metal-Oxide-Semiconductor Field-Effect Transistor
GBWP	Gain-Bandwidth Product
Op-Amps	Operational Amplifiers
DTT	Digital Terrestrial Television
LTE	Long-Term Evolution
GSM	Global System for Mobile communication
LoRa	Long Range Wide Area
PVT	Process Voltage Temperature
OTA	Operational Transconductance Amplifier
SRF	Self-Resonant Frequency
MIM	Metal Insulator Metal
GBW	Gain Bandwidth
PSS	Periodic Steady State
IIP3	Input Third-Order Intercept Point
BW	Bandwidth
NF	Noise Figure
OTV	Oxide Thickness Variation
WKF	Metal Gate Work Function Fluctuations
RDF	Random Dopant Fluctuations
LWR	Line-Width Roughness
DR	Dynamic Range
FoM	Figure of Merit
